# The application of artificial intelligence for Rapid On-Site Evaluation during flexible bronchoscopy

**DOI:** 10.3389/fonc.2024.1360831

**Published:** 2024-03-11

**Authors:** Shuang Yan, Yongfei Li, Lei Pan, Hua Jiang, Li Gong, Faguang Jin

**Affiliations:** ^1^ Department of Pulmonary and Critical Care Medicine, Tangdu Hospital, Air Force Medical University, Xi’an, China; ^2^ Xi'an High-tech Institute, Xi’an, China; ^3^ Department of Pathology, Tangdu Hospital, Air Force Medical University, Xi’an, China

**Keywords:** rapid on-site evaluation, transbronchial biopsy, lung cancer, flexible bronchoscopy, deep convolutional neural network

## Abstract

**Background:**

Rapid On-Site Evaluation (ROSE) during flexible bronchoscopy (FB) can improve the adequacy of biopsy specimens and diagnostic yield of lung cancer. However, the lack of cytopathologists has restricted the wide use of ROSE.

**Objective:**

To develop a ROSE artificial intelligence (AI) system using deep learning techniques to differentiate malignant from benign lesions based on ROSE cytological images, and evaluate the clinical performance of the ROSE AI system.

**Method:**

6357 ROSE cytological images from 721 patients who underwent transbronchial biopsy were collected from January to July 2023 at the Tangdu Hospital, Air Force Medical University. A ROSE AI system, composed of a deep convolutional neural network (DCNN), was developed to identify whether there were malignant cells in the ROSE cytological images. Internal testing, external testing, and human-machine competition were used to evaluate the performance of the system.

**Results:**

The ROSE AI system identified images containing lung malignant cells with the accuracy of 92.97% and 90.26% on the internal testing dataset and external testing dataset respectively, and its performance was comparable to that of the experienced cytopathologist. The ROSE AI system also showed promising performance in diagnosing lung cancer based on ROSE cytological images, with accuracy of 89.61% and 87.59%, and sensitivity of 90.57% and 94.90% on the internal testing dataset and external testing dataset respectively. More specifically, the agreement between the ROSE AI system and the experienced cytopathologist in diagnosing common types of lung cancer, including squamous cell carcinoma, adenocarcinoma, and small cell lung cancer, demonstrated almost perfect consistency in both the internal testing dataset (κ  = 0.930 ) and the external testing dataset (κ  = 0.932 ).

**Conclusions:**

The ROSE AI system demonstrated feasibility and robustness in identifying specimen adequacy, showing potential enhancement in the diagnostic yield of FB. Nevertheless, additional enhancements, incorporating a more diverse range of training data and leveraging advanced AI models with increased capabilities, along with rigorous validation through extensive multi-center randomized control assays, are crucial to guarantee the seamless and effective integration of this technology into clinical practice.

## 1 Introduction

Lung cancer is the world’s leading cause of cancer death (1). Despite improvements in survival for most cancer types over the last several decades, lung cancer lags behind, mainly because it is initially asymptomatic and typically discovered at advanced stages (2, 3). Currently, flexible bronchoscopy (FB) is the most commonly-used modality for the diagnosis of lung lesions (4), and the transbronchial biopsy has been typically performed as a safe and effective procedure to sample tissues to differentiate malignant from benign lesions and staging of lung cancer (5). However, whether the operator has got sufficient and satisfactory specimens during the biopsy procedure is unknown. In recent years, different methods have been developed to improve the adequacy of specimens obtained through the biopsy procedure, such as the Rapid On-Site Evaluation (ROSE) (6–8). ROSE was used especially to assist clinicians in the diagnosis of lesions that are not directly visible at a standard bronchoscopic airway examination, such as those located in the mediastinum or the pulmonary parenchyma (9). With ROSE, the specimen is prepared and stained after the biopsy procedure, allowing for immediate cytopathologic evaluation and feedback regarding specimen adequacy and potential diagnosis (10). Recent studies demonstrated that ROSE during the bronchoscopy can result in a low rate of non-diagnostic sampling and yield a high agreement between the on-site and final pathological evaluation (11–13). Malignant results of ROSE may be useful to facilitate an early clinical decision.

However, the shortage of cytopathologists limits the wide use of ROSE in many institutes worldwide, especially in institutes of underdeveloped countries. On the other hand, with the development of digital imaging and the rapid progress in machine learning technique, automatic pathological image assessments with artificial intelligence (AI) has become available (14, 15), thus making it possible to replace manual ROSE with the AI system, which would promote the utility of ROSE and improve the diagnostic yield of FB. To the best of our knowledge, only a few studies have explored the application of AI in ROSE (16, 17), and none have investigated the application of AI-based ROSE in the diagnosis of lung lesions. Therefore, in this study, we developed a ROSE AI system to access the cytological images obtained from the transbronchial biopsy, evaluated the clinical performance of the ROSE AI system, and discussed the possibility of replacing manual ROSE with the ROSE AI system.

## 2 Materials and methods

### 2.1 Data collection

**Figure 1:**
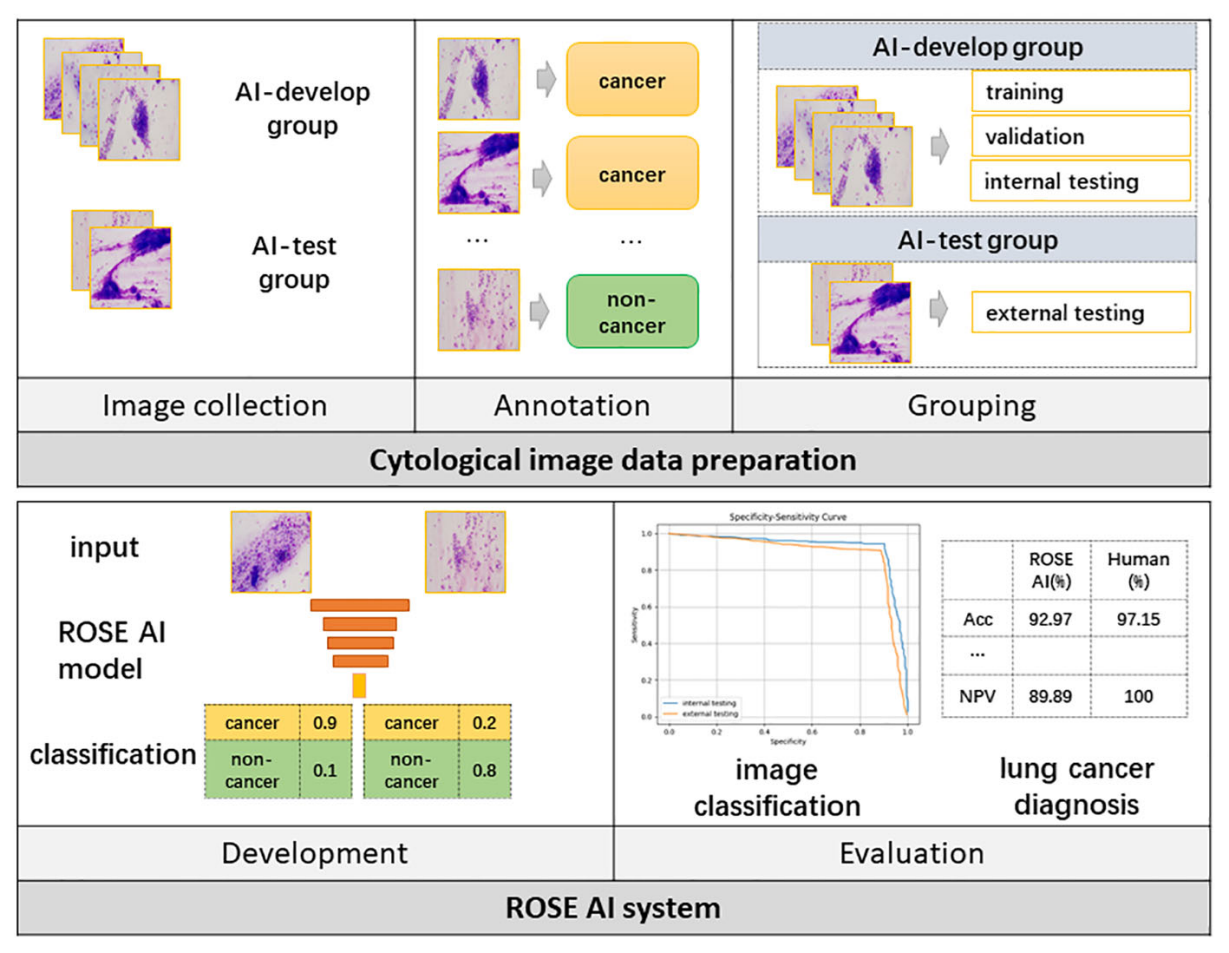
The flow chart of the study design. AI, Artificial Intelligence; ROSE, Rapid On-Site Evaluation; Acc, Accuracy.

From January to July 2023, 6357 ROSE cytological images from 721 patients who underwent transbronchial biopsy were collected at the Tangdu Hospital, Air Force Medical University. Among the 721 patients who underwent bronchoscopic biopsy, the lesions of 390 patients were identified through direct visualization under bronchoscopy, while the lesions of 323 patients were detected using radial-endobronchial ultrasound with guide sheath (r-EBUS-GS). In 8 cases, ultrasound did not detect the lesions, and the blind biopsy approach was employed. 5176 ROSE cytological images from 576 patients, which were collected between January and May 2023, were used to develop the ROSE AI system and test the system internally, while 1181 images from 145 patients collected from June to July 2023 were used to test the ROSE AI system externally. The former was called the AI-develop group, while the latter was called the AI-test group. The flow chart of the study design is shown in **Figure 1**.

The study was approved by the regional ethics committee of Tangdu Hospital, Air Force Medical University (TDLL-202311-03).

### 2.2 ROSE slides acquisition

**Patient preparation:** All patients received local anesthesia using 2% lidocaine spray before undergoing FB. A preoperative assessment was conducted to rule out contraindications. The FB used were Olympus BF-260, BF-P260, and BF-P290. Different bronchoscopes were used according to their availability. **Transbronchial biopsy:** First, a flexible bronchoscope provided visualization and access to the bronchus. Subsequently, a gentle but precise tissue sampling was performed by opening and closing the biopsy forcesp, capturing a small segment of the lesion. Five to six biopsy specimens were taken from each lesion site. **ROSE smear:** ROSE slide preparation was conducted by a proficient and professionally trained cytotechnologist based on methods reported in the literature (18) for each specimen. Each biopsy specimen was spread in a concentric circle with a diameter of 1 centimeter on a sterile cytology slide. The slides were then air-dried and stained using a Diff-Quik stain kit (immersion in A solution for 30 seconds, rinsing with phosphate-buffered saline (PBS), immersion in B solution for 20 seconds, rinsing with PBS), and finally observed under an Olympus BX43 microscope. The remaining tissue after preparation of the ROSE slides was placed in tissue fixative fluid and sent for pathological examination.

### 2.3 ROSE cytological image acquisition

All available ROSE slides of the included patients were photographed with a camera (Olympus DP74) mounted on the microscope (Olympus BX43) at 200X magnification. All the images were labeled by an experienced cytopathologist as cancer or non-cancer to indicate whether there exist malignant cells in the images under the guidance of the final pathological diagnoses. To ensure the accuracy of the annotation results, the label for one image was accepted only when the annotation by ROSE cytopathologist was consistent with the final pathological diagnosis. Otherwise, the image was removed from the dataset.

After data annotation, all the images in the AI-develop group were mixed, and randomly assigned to training dataset, validation dataset, or internal testing datasets with a probability of 0.8, 0.1, and 0.1 respectively. All the images in the AI-test group were considered as the external testing dataset. The details for the datasets are shown in **Table 1**.

(1) The training dataset, which includes 2738 images from 346 patients labeled with cancer, and 1368 images from 229 patients labeled with non-cancer, was used to train the ROSE AI system.
(2) The validation dataset, which includes 341 images from 215 patients labeled with cancer, and 203 images from 132 patients labeled with non-cancer, was used to validate the model to select the optimal AI model and hyperparameters.
(3) The internal testing dataset, which includes 339 images from 212 patients labeled with cancer, and 187 images from 125 patients labeled with non-cancer, was used to test the ROSE-AI system internally.
(4) The external testing dataset, which includes 866 images from 98 patients labeled with cancer, and 315 images from 47 patients labeled with non-cancer, was used to test the model externally.

**Table 1:** Patient demographics and clinical information.

dataset	image		patient				
	cancer	non-cancer	diagnosis		gender		age (year)
			cancer	non-cancer	male	female	
train	2738	1368	346	229	395	180	60.07 ± 12.04
validation	341	203	215	132	245	102	60.22 ± 12.04
internal testing	339	187	212	125	226	111	60.30 ± 12.18
external testing	866	315	98	47	105	40	61.64 ± 10.39

Both the internal and external testing dataset were also used to compare the performance of the ROSE AI system with cytopathologists.

### 2.4 Development of the ROSE AI system

**Design ROSE AI model.** The basic function of the ROSE AI system is to verify whether a ROSE cytological image contains malignant cells or not. We trained an AI classification model to identify whether there existed malignant cells in a given ROSE cytological image. Several classification models using deep convolutional neural network (DCNN) have been trained on the training dataset, and the ResNet101 (19) (implemented with Pytorch 1.13.1), which achieved the best performance on the validation dataset was selected finally.

**Train ROSE AI model.** The ROSE AI model was trained on two NVIDIA 3090Ti GPUs with the training dataset. All images in the training dataset were resized into 1024x1024 pixels before feeding into the classification model. The data augmentation strategies, which include random flipping, random rotation, and random center cropping, were used to increase the amount of training data to boost the training performance of the system. We used the cross entropy loss function to provide feedback on the model. Label smoothing, a regularization technique, was used to prevent overfitting and improve the generalization performance. The AI ROSE model was trained for a total cycle of 200 epochs (Adam optimizer with a learning rate of 0.0001 and batch size of 8). After each epoch, the trained model was tested on the validation dataset. The model which achieved the highest accuracy on the validation dataset was selected as the final AI model.

### 2.5 Evaluation on the clinical performance of the ROSE AI system

The performance of identifying lung malignant cells of the ROSE AI system was firstly accessed on the internal testing dataset and the external testing dataset. The classification result of the ROSE AI system for each image was validated with the annotation. And then the accuracy, sensitivity, specificity, PPV, and NPV were analyzed. An experienced cytopathologist was asked to identify the existence of malignant cells in the same dataset, without details regarding the clinical characteristics, endoscopic features, and pathological results presented. The performance of the cytopathologist was compared with our ROSE AI system.

Then the ability of the ROSE AI system to diagnose lung cancer based on cytological images was also evaluated. The ROSE AI system would diagnose a patient with cancer when at least one cytological image was identified containing malignant cells by the ROSE AI system. The experienced cytopathologist provided a comprehensive diagnosis based on all ROSE cytological images of the same patient. Both the judgment of the ROSE AI system and the cytopathologist for each patient were validated with the annotation, and the accuracy, sensitivity, specificity, PPV, and NPV were analyzed.

### 2.6 Statistical analysis

The accuracy, sensitivity, specificity, PPV, and NPV were computed to measure the performance of the ROSE AI system and cytopathologist at the per-image level and per-patient level using Python (version 3.9.13). The consistency of the ROSE AI system and the cytopathologist was evaluated with the Kappa test using SPSS (version 26.0).

## 3 Results

### 3.1 Patient characteristics

From January 2023 to July 2023, 721 patients (shown in **Table 1**) diagnosed with lung lesions based on chest computed tomography (CT) underwent FB with ROSE. A total of 751 lesions were biopsied from 721 patients. Among these, 582 (77.50%) lesions were located peripherally, while 169 (22.50%) lesions were located centrally. 6357 ROSE images were taken for all the patients, with 8.82 ± 3.20 images for each patient. The mean age of the patients was 60.40 ± 11.74, and 69.49% of patients (501) were male. Among all the patients, 61.72% of patients (445) were diagnosed with cancer, and 38.28% of patients (276) were diagnosed with non-cancerous conditions. The patient characteristics in each dataset were analyzed (shown in **Table 1**).

Recognizing the significance of assessing the ROSE AI system’s robustness across diverse settings and patient populations, we conducted a detailed analysis focusing on patients in the AI-test group. Among the 145 patients examined, 137 underwent biopsy for a single lesion, while 7 patients underwent biopsies for two lesions, and 1 patient underwent biopsies for three lesions. Of the total 154 lesions examined, 114 (74.03%) were located peripherally, while 40 (25.97%) were located centrally. Furthermore, among the 145 patients, 79 (54.48%) had lesions identified through direct visualization under bronchoscopy, while 63 (43.45%) had lesions detected using radial-endobronchial ultrasound with a guide sheath (r-EBUS-GS). In 3 (2.07%) cases, ultrasounds failed to detect the lesions, necessitating the use of a blind biopsy approach.

The final pathological diagnoses of patients were presented in **Table 2**. In the AI-develop group, 229 patients were diagnosed as non-cancerous, 108 patients with squamous cell carcinoma, 109 patients with adenocarcinoma, and 59 patients with small cell lung cancer. Additionally, specimens from 55 patients in the AI-develop group were histopathologically diagnosed as malignant, but a specific subtype was not identified due to the lack of follow-up immunohistochemistry or unavailable follow-up information. These cases were categorized as “no specific subtype.” Furthermore, 16 patients in the AI-develop group were categorized as “others”, including adenoid cystic carcinoma (1 patient), B-cell lymphoma (1 patient), adenocarcinoma combined with small cell lung cancer (1 patient), breast cancer metastasis (1 patient), mesothelioma (3 patients), sarcoma (1 patient), adenosquamous carcinoma (2 patients), large cell carcinoma (1 patient), squamous cell carcinoma combined with neuroendocrine carcinoma (1 patient), cervical cancer lung metastasis (1 patient), renal malignant tumor metastasis (1 patient), thyroid cancer metastasis (1 patient), and classical Hodgkin lymphoma (1 patient). The final pathological diagnoses for AI-test group patients were also presented in **Table 2**, indicating 47 non-cancerous, 31 squamous cell carcinoma, 33 adenocarcinoma, 19 small cell lung cancer, and 15 no specific subtype.

**Table 2:** Final pathological diagnoses of patients.

	pathological diagnosis	AI-develop group			AI-test group
		train	validation	internal testing	external testing
cancer	squamous cell carcinoma	108	69	56	31
	adenocarcinoma	109	63	74	33
	small cell lung cancer	59	42	41	19
	others^a)^	16	9	9	0
	no specific subtype^b)^	54	32	32	15
non-cancer		229	132	125	47

^a)^ “others” includes: adenoid cystic carcinoma (1 patient), B-cell lymphoma (1 patient), adenocarcinoma combined with small cell lung cancer (1 patient), breast cancer metastasis (1 patient), mesothelioma (3 patients), sarcoma (1 patient), adenosquamous carcinoma (2 patients), large cell carcinoma (1 patient), squamous cell carcinoma combined with neuroendocrine carcinoma (1 patient), cervical cancer lung metastasis (1 patient), renal malignant tumor metastasis (1 patient), thyroid cancer metastasis (1 patient), classical Hodgkin lymphoma (1 patient).

^b)^ The specimens obtained from the patients were diagnosed as malignant through histopathology, but a specific subtype was not identified. Follow-up immunohistochemistry was either not conducted, or the follow-up information was unavailable.

### 3.2 Performance of the ROSE AI system in image classification

**Figure 2:**
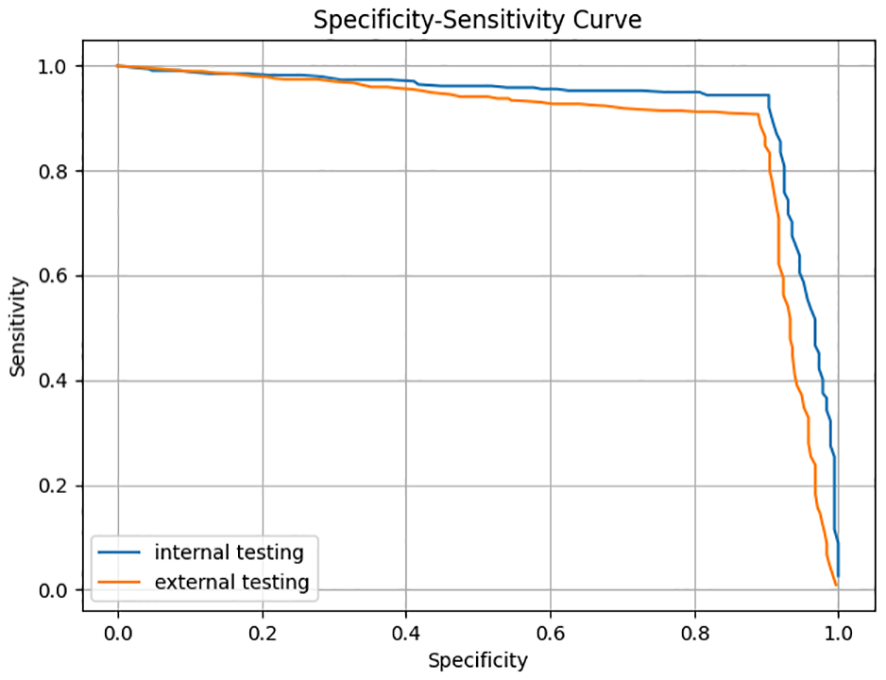
Specificity-sensitivity curve of image classification.

The trained AI ROSE classification model was evaluated on the internal testing dataset and the external testing dataset. For each ROSE image, the ROSE AI classification model determined whether it contained malignant cells or not. The performance of ROSE AI classification was measured with the accuracy, sensitivity, specificity, PPV, and NPV. The specificity-sensitivity curves on both internal and external testing datasets are shown in **Figure 2**.

A classification confidence threshold of 0.5 was selected to compute the final accuracy, sensitivity, specificity, PPV, and NPV on both internal and external testing datasets, the results are shown in **Table 3**. The accuracy, sensitivity, specificity, PPV, and NPV of the ROSE AI classification in classifying cancer images were 92.97%, 94.40%, 90.37%, 94.67%, and 89.89% respectively on the internal testing dataset, and 90.26%, 90.76%, 88.89%, 95.74%, and 77.78% respectively on the external testing dataset. Meanwhile, the performance of an experienced cytopathologist in classifying cancer images was 97.15% (accuracy), 100.00% (sensitivity), 91.98% (specificity), 95.76% (PPV), and 100.00% (NPV) on the internal testing dataset, and 94.83% (accuracy), 94.69% (sensitivity), 95.24% (specificity), 98.20% (PPV), and 86.71% (NPV) on the external testing dataset. The consistency between the ROSE AI system and the experienced cytopathologist was good ( κ=0.798 on the internal testing dataset, κ=0.673 on the external testing dataset). Collectively, these results indicated that the ROSE AI classification model can identify whether there were malignant cells in ROSE cytological images with high accuracy and substantial robustness.

**Table 3:** Performance of the ROSE AI system in image classification.

dataset		accuracy (%)	sensitivity (%)	specificity (%)	PPV (%)	NPV (%)
internal testing	ROSE^a)^ AI^b)^	92.97	94.40	90.37	94.67	89.89
	human	97.15	100.00	91.98	95.76	100.00
external testing	ROSE^a)^ AI^b)^	90.26	90.76	88.89	95.74	77.78
	human	94.83	94.69	95.24	98.20	86.71

^a)^ ROSE, Rapid On-Site Evaluation; ^b)^AI, Artificial Intelligence.

### 3.3 Performance of the ROSE AI system in identifying lung cancer

We next evaluated the performance of the ROSE AI system in identifying lung cancer in ROSE cytological images at the per-patient level. A patient was diagnosed with lung cancer by the ROSE AI system when at least one ROSE cytological image of the patient was classified as a cancer image (with a confidence threshold of 0.55). Representative predictions of the ROSE AI system are shown in **Table 4**. The results showed that the ROSE AI system identified lung cancer with an accuracy of 89.61%, sensitivity of 90.57%, specificity of 88.00%, PPV of 92.75%, and NPV of 84.62% on the internal testing dataset, and accuracy of 87.59%, sensitivity of 94.90%, specificity of 72.34%, PPV of 87.74%, and NPV of 87.18% on the external testing dataset.

**Table 4:** Performance of the AI ROSE system in identifying lung cancer.

dataset		accuracy (%)	sensitivity (%)	specificity (%)	PPV (%)	NPV (%)
internal testing	ROSE^a)^ AI^b)^	89.61	90.57	88.00	92.75	84.62
	human	97.03	100.00	92.00	95.50	100.00
external testing	ROSE^a)^ AI^b)^	87.59	94.90	72.34	87.74	87.18
	human	95.17	96.94	91.49	95.96	93.48

^a)^ ROSE, Rapid On-Site Evaluation; ^b)^ AI, Artificial Intelligence.

We also asked an experienced cytopathologist to diagnose the patients based on the same ROSE cytological images dataset. The experienced cytopathologist provided a comprehensive diagnosis based on all ROSE cytological images of one patient. The accuracy, sensitivity, specificity, PPV, and NPV of the experienced cytopathologist in diagnosing lung cancer were 97.03%, 100.00%, 92.00%, 95.50%, and 100.00% respectively on the internal testing dataset, and 95.17%, 96.94%, 91.49%, 95.96%, and 93.48% respectively on the external testing dataset. The consistency between the ROSE AI system and experienced cytopathologist was also analyzed with the Kappa test. The Kappa values on internal and external testing datasets were 0.738 and 0.618, indicating good consistency between the two diagnoses.

### 3.4 Disparities between the ROSE AI system and cytopathologist in image classification

Despite the overall good consistency observed between the ROSE AI system and the experienced cytopathologist, there were still significant instances where interpretations diverged between the two. To further scrutinize the factors contributing to disparities between the ROSE AI system and cytopathologist’s interpretations, we conducted an analysis specifically focusing on cases where inconsistencies between the ROSE AI system and cytopathologists emerged. The detailed findings are presented in **Table 5**. Examining **Table 5** revealed that in the internal testing dataset, the predominant instances of discrepancies between the ROSE AI system and pathologists were associated with diagnoses such as “non-cancer” (15.51%), “no specific subtype” (8.89%), “small cell lung cancer” (8.77%), and others (7.69%). Similarly, in the external testing dataset, the most notable instances of discrepancies encompassed “no specific subtype” (22.73%), “non-cancer” (15.87%), and “small cell lung cancer” (13.59%).

**Table 5:** Statistical analysis of inconsistencies between the ROSE AI system and pathological interpretations.

dataset		non-cancer	squamous cell carcinoma	adenocarcinoma	small cell lung cancer	others^c)^	no specific subtype^d)^
internal testing	N (total)^a)^	187	94	130	57	13	45
	N (inconsistent)^b)^	29	2	7	5	1	4
	percentage (%)	15.51	2.13	5.38	8.77	7.69	8.89
external testing	N (total)^a)^	315	256	316	184	0	110
	N (inconsistent)^b)^	50	32	30	25	–	25
	percentage (%)	15.87	12.50	9.49	13.59	–	22.73

^a)^ The total number of images from patients with certain final pathological diagnoses.

^b)^ The total number of images where the discrepancies between the ROSE AI system and cytopathologists occur.

^c)^ “others” includes: adenoid cystic carcinoma (1 patient), B-cell lymphoma (1 patient), mesothelioma (2 patients), sarcoma (1 patient), squamous cell carcinoma combined with neuroendocrine carcinoma (1 patient), cervical cancer lung metastasis (1 patient), thyroid cancer metastasis (1 patient), classical Hodgkin lymphoma (1 patient).

^d)^ The specimens obtained from the patients were diagnosed as malignant through histopathology, but a specific subtype was not identified. Follow-up immunohistochemistry was either not conducted, or the follow-up information was unavailable.

### 3.5 Performance of the ROSE AI system in identifying various types of tumors as malignant

To further evaluate the efficacy of the ROSE-AI system, we conducted a detailed analysis of its performance in identifying various types of tumors as malignant, with a particular focus on non-primary lung tumors and less prevalent malignant lung tumors. The results are comprehensively outlined in **Table 6**. Patients with final pathological diagnoses of squamous cell carcinoma, adenocarcinoma, and small cell lung cancer were classified under the “common” group. Meanwhile, those with less prevalent malignant lung tumors and non-primary lung tumors were grouped under “others”, and patients pathologically diagnosed as malignant but without a specific subtype were categorized under “no specific subtype.” Since all patients in these three groups were diagnosed with malignant tumors, sensitivity equaled accuracy, and specificity, PPV, and NPV were 0%, 100%, and 0%, respectively. Consequently, we solely presented accuracy as the performance metric in **Table 6**. In the internal testing dataset, the accuracy for the “common,” “others,” and “no specific subtype” groups was 91.81%, 88.89%, and 84.38%, respectively. For the external testing dataset, no patients were diagnosed with the “others” category, and as such, no accuracy is reported for this group. The accuracy for the remaining two groups in the external testing dataset was 96.39% for the “common” group and 86.67% for the “no specific subtype” group. The Kappa test was subsequently administered to the “common” group in both the internal and external testing datasets, yielding Kappa values of 0.930 and 0.932, respectively. These results suggest almost perfect consistency between the diagnoses provided by the ROSE AI system and those of experienced cytopathologist for common lung malignant tumors.

**Table 6:** Comparison on the performance of the AI ROSE system in identifying different tumors as malignant.

dataset	accuracy (%)		
	common^a)^	others^b)^	no specific subtype^c)^
internal testing	91.81	88.89	84.38
external testing	96.39	–	86.67

^a)^ “common” includes: squamous cell carcinoma, adenocarcinoma, and small cell lung cancer;

^b)^ “others” includes: adenoid cystic carcinoma (1 patient), B-cell lymphoma (1 patient), mesothelioma (2 patients), sarcoma (1 patient), squamous cell carcinoma combined with neuroendocrine carcinoma (1 patient), cervical cancer lung metastasis (1 patient), thyroid cancer metastasis (1 patient), classical Hodgkin lymphoma (1 patient).

^c)^ The specimens obtained from the patients were diagnosed as malignant through histopathology, but a specific subtype was not identified. Follow-up immunohistochemistry was either not conducted, or the follow-up information was unavailable.

## 4 Discussion

In this study, we developed a novel ROSE AI system to render ROSE during transbronchial biopsy. The ROSE AI system showed promising performance in identifying whether there exist lung malignant cells in the ROSE cytological images, and in diagnosing lung cancer based on all ROSE cytological images of the patient. The results of this study indicated that the diagnostic performance of the ROSE AI system could be comparable to that of experienced cytopathologists. To the best of our knowledge, this study was the first to establish a deep learning-based classification system for identifying lung malignant cells in ROSE during transbronchial biopsy, which might improve the diagnostic performance of FB.

ROSE can evaluate the diagnostic adequacy of the specimens obtained with transbronchial biopsy instantly and thus is pivotal to improve the diagnostic yield of FB and help to avoid unnecessary repeat biopsy (10, 20–22). However, the lack of cytopathologists in many hospitals has refrained from the wide use of ROSE. Recently, with the rapid development of AI, especially the fast progress in deep learning technique, the application of AI in digital pathology have made great success (23–26). It may be promising to utilize AI to render ROSE during transbronchial biopsy. However, to our knowledge, there have been no studies implementing AI in evaluating the diagnostic adequacy of transbronchial biopsy for diagnosing lung cancer. In this paper, we developed a ROSE AI system, which achieved an accuracy of 92.97% and 90.26% in identifying the existing of lung malignant cells in ROSE cytological image in the internal testing dataset and external testing dataset respectively, and is comparable with the performance of experienced cytopathologists. The accuracy only decreased slightly on the external testing dataset compared with that on the internal testing dataset, which indicated the trained ROSE AI classification model generalized well and was accurate and robust. Therefore, the ROSE AI system exhibits potential for assessing the adequacy of biopsy specimens during FB in clinical settings.

Furthermore, the ROSE AI system also showed promising performance in identifying lung cancer, with an accuracy of 89.61% and 87.59% on the internal testing dataset and external testing dataset respectively, which was slightly inferior than that of experienced cytopathologists (97.03% on the internal testing dataset, and 95.17% on external testing dataset). It was reported that the ROSE diagnostic accuracy of the junior and mid-level endoscopists for superficial esophageal squamous cell carcinoma was less than 80% (27). Our ROSE AI system achieved better diagnostic performance than the junior and mid-level endoscopists. Besides, the sensitivity of our ROSE AI system was 90.57% and 94.90% on the internal testing dataset and external testing dataset respectively, which means less than 10% of patients with lung cancer were misdiagnosed by the ROSE AI system. Hence, our ROSE AI system has the potential to be implemented in clinical settings to support endoscopists in enhancing the diagnostic yield of FB.

Compared to pathologists, the performance of the ROSE AI system was slightly inferior in image classification. **Table 5** indicated that when the patient’s final pathological diagnosis was “non-cancer,” “no specific subtype,” or “small cell lung cancer,” there was a higher likelihood of discrepancies between the ROSE AI system’s interpretation and that of pathologists. Conversely, when the patient’s diagnosis was “squamous cell carcinoma” or “adenocarcinoma,” the agreement between the two was higher. This suggests that, compared to cases diagnosed as “non-cancer,” “no specific subtype,” or “small cell lung cancer,” the current ROSE AI system demonstrates more accurate interpretations for cases diagnosed as “squamous cell carcinoma” or “adenocarcinoma.” In this study, cases diagnosed as “non-cancer” encompassed various conditions such as infections and benign nodules, resulting in greater diversity in the images. This increased diversity might make it more challenging for the ROSE AI system to diagnose such cases accurately. This also suggests that enhancing the diversity of cytopathological images for training could be a potential improvement to enhance the performance of the ROSE AI system.

Moreover, we conducted an in-depth analysis of the ROSE AI system’s performance in distinguishing various types of tumors as malignant, with a specific emphasis on non-primary lung tumors and less prevalent malignant lung tumors. As depicted in both **Tables 4**, **6**, when compared to its overall performance in identifying all lung tumors as malignant, the ROSE AI system exhibits a higher accuracy in identifying common lung tumors as malignant—encompassing squamous cell carcinoma, adenocarcinoma, and small cell lung cancer (89.61% vs. 91.81% in the internal testing dataset and 87.59% vs. 96.39% in the external testing dataset). The consistency between the ROSE AI system’s diagnoses and those of experienced cytopathologists reached nearly perfect levels for common lung tumors, with Kappa values of 0.930 and 0.932 in the internal and external testing datasets, respectively. This implies that the ROSE AI system excels in identifying common lung tumors as malignant. However, for less prevalent malignant lung tumors and non-primary lung tumors, the accuracy was slightly lower, standing at 88.89% in the internal testing dataset. This discrepancy may be attributed to the scarcity of training data, as the training dataset comprised 1438 images for common lung tumors compared to only 127 images for less prevalent malignant lung tumors and non-primary lung tumors.

Even though our ROSE AI system has achieved promising results, several limitations still exist in this study. Firstly, it is essential to acknowledge that ROSE cannot substitute the comprehensive assessment offered by a final pathological examination. Achieving perfect consistency (100%) between ROSE and pathological examination poses a significant challenge. Moreover, it’s important to note that ROSE cannot offer detailed information on tumor subtyping and staging. Secondly, the study design employed was a single-center retrospective approach. This choice, characteristic of a single-center study, raised concerns about the potential limitation in data diversity, thereby constraining a thorough evaluation of the ROSE AI system’s performance. Furthermore, being retrospective in nature, the research cannot assure the completeness of data. An illustrative example is the unavailability of precise statistics regarding lesion sizes, hindering our ability to conduct a detailed assessment of the ROSE AI system’s efficacy across lesions of different sizes. In the future, multi-center prospective randomized controlled clinical trials are needed to assess the potential role of ROSE AI system in clinical practice. Finally, our ROSE AI system demonstrated performance comparable to, albeit slightly below, that of experienced cytopathologists. The observed limitation could stem from the insufficient diversity in the training data, particularly in cases where patients were diagnosed as “non-cancer.” To address this, we intend to expand our collection of ROSE cytological images, aiming to augment the diversity of the training data and consequently improve the performance of our ROSE AI system. Additionally, exploring novel deep learning based classification network architectures, specifically those tailored for small samples, represents a promising avenue for enhancing the capabilities of the ROSE AI system.

In the integration of the ROSE AI system into clinical applications, several ethical considerations demand attention. Firstly, the inherent biases within AI algorithms, derived from the data they are trained on, may result in disparate healthcare outcomes among various demographic groups. Besides, there exist risks of overreliance on algorithms which may lead to potential errors or misdiagnoses. Therefore, ensuring the rigorous validation and continuous monitoring of the ROSE AI system is paramount to uphold patient safety. Conducting more extensive and larger multi-center randomized control assays becomes essential to validate its safety and reliability before widespread use in medical applications. Secondly, the reliance of AI diagnostic systems on vast amounts of sensitive patient data underscores the critical need for robust data privacy regulations and cybersecurity measures. Finally, the integration of the ROSE AI system in medicine presents complex regulatory and legal challenges, including issues of responsibility attribution in cases of errors or adverse outcomes. It is imperative to establish ethical frameworks and guidelines that prioritize patient welfare, autonomy, and informed consent, while also addressing the broader societal implications of AI adoption in healthcare.

## 5 Conclusion

We developed a ROSE AI system to assist endoscopists in rendering ROSE during transbronchial biopsy. Our ROSE AI system showed comparable performance to experienced cytopathologists in identifying the existence of malignant cells in ROSE cytological images, indicating its potential role in evaluating the adequacy of biopsy specimens during FB in clinical practice. Besides, the good performance of our ROSE AI system in identifying lung cancer indicated its potential deployment in clinical settings to aid endoscopists in enhancing the diagnostic yield of FB. Nevertheless, the ROSE AI system requires additional validation, considering the limitations of this study being a single-center retrospective analysis. Furthermore, the current performance of the ROSE AI system remains subpar compared to experienced cytopathologists. It is crucial to emphasize that, at this stage, the ROSE AI system cannot serve as a substitute for experienced cytopathologists in clinical practice. Moreover, a comprehensive examination of ethical considerations is essential before advocating for the broader implementation of the ROSE AI system in clinical applications. This scrutiny should encompass issues such as bias and fairness within the AI algorithm, patient safety, data privacy and security, as well as regulatory compliance and legal implications, necessitating thorough and rigorous attention.

## Data availability statement

The raw data supporting the conclusions of this article will be made available by the authors, without undue reservation.

## Ethics statement

The studies involving humans were approved by Regional Ethics Committee of Tangdu Hospital, Air Force Medical University. The studies were conducted in accordance with the local legislation and institutional requirements. The participants provided their written informed consent to participate in this study.

## Author contributions

SY: Conceptualization, Data curation, Formal analysis, Investigation, Methodology, Software, Validation, Visualization, Writing – original draft, Writing – review & editing. YL: Conceptualization, Data curation, Formal analysis, Investigation, Methodology, Software, Validation, Visualization, Writing – original draft, Writing – review & editing. LP: Conceptualization, Data curation, Funding acquisition, Project administration, Supervision, Writing – review & editing. HJ: Data curation, Writing – review & editing. LG: Data curation, Writing – review & editing. FJ: Conceptualization, Funding acquisition, Project administration, Supervision, Writing – review & editing.
